# The Urea–Creatinine Ratio as Marker of Catabolism Is Affected by Continuous Renal Replacement Therapy

**DOI:** 10.3390/diagnostics15111408

**Published:** 2025-06-01

**Authors:** Andreas Markl-Le Levé, Petra Hillinger, Simon Woyke, Marco Ronzani, Stefan Schmid, Janett Kreutziger, Christopher Rugg

**Affiliations:** Department of Anaesthesiology and Critical Care Medicine, Medical University of Innsbruck, Anichstrasse 35, 6020 Innsbruck, Austria

**Keywords:** urea–creatinine ratio, urea, creatinine, catabolism, acute kidney injury, continuous renal replacement therapy

## Abstract

**Background:** An elevated urea–creatinine ratio (UCR) is used as a surrogate for catabolism and elevated protein metabolism in critically ill patients. This study investigated the effect of continuous renal replacement therapy (CRRT) on UCR. **Methods:** In this retrospective single-centre study, ICU patients from 2011 to 2022 with an ICU stay >2 days before CRRT and a CRRT duration of ≥4 days were included. Patients were grouped by UCR at CRRT initiation into high (UCR ≥ 75 mg/dL:mg/dL) and low groups and compared to matched controls not requiring CRRT. Propensity score matching considered age, sex, bodyweight, SAPS3, SOFA score, and UCR values on baseline and pre-baseline days. **Results:** In the high UCR group, UCR significantly decreased after CRRT initiation, reaching a significant difference from controls on day 2 (85.0 [IQR: 69.5–96.4] vs. 94.4 [IQR: 83.0–115.2]; *p* = 0.036) and falling below the threshold of 75 by day 3. In the low group, UCR increased post-CRRT initiation, but was less pronounced than in controls, with significant differences on day 1 (44.0 [IQR: 34.2–59.8] vs. 40.6 [IQR: 32.1–52.5]; *p* = 0.024). **Conclusions:** CRRT significantly affects UCR in critically ill patients, showing a marked decrease when compared to matched controls.

## 1. Introduction

The urea–creatinine ratio (UCR) serves as a biochemical marker of muscle catabolism in critically ill patients [1,2,3]. Muscle catabolism likely increases urea production, while the progressive loss of muscle mass reduces creatinine generation, hence leading to an increased UCR [2]. In a study conducted by Haines et al., a value of 141 mmol/L:mmol/L (≙75 mg/dL:mg/dL) was accompanied by a prolonged ICU stay and a decreased psoas cross-sectional muscle area at the L4 level, therefore indicating an increased protein metabolism [1]. An elevated UCR has been associated not only with a reduction in muscle mass [1], but also with increased urea production and subsequent urea-induced osmodiuresis, leading to a substantial loss of electrolyte-free water and consecutive hypernatremia [4,5]. It was mentioned as a possible marker to monitor catabolism and muscle mass in a recent expert recommendation [6]. High protein intake during the early ICU phase may lead to an elevated UCR, reflecting not only catabolism but also increased exogenous protein supplementation [7]. Furthermore, an increased UCR has been associated with an increased risk of death during prolonged ICU stay [8].

Since both urea and creatinine are primarily excreted renally, it may seem intuitive to assume that renal function has minimal impact on the UCR. However, besides other factors potentially increasing UCR (e.g., gastrointestinal bleeding [9], decreased effective blood volume [10]), severe acute kidney injury (AKI) tended to lower the UCR in the study conducted by Haines et al. [1]

The impact of continuous renal replacement therapy (CRRT) on the UCR has not yet been thoroughly explored. Urea and creatinine are both small molecules, and their elimination rates should be quite comparable [11]. CRRT filter kinetic studies have also shown no significant difference in the clearance of urea and creatinine, supporting the use of the UCR to monitor the metabolic state of critically ill patients, even during CRRT [12,13,14,15].

This study aims to analyze the effects of CRRT on the UCR and provide new insights to enhance understanding in this field.

## 2. Materials and Methods

This retrospective study was approved by the Ethics Committee of the Medical University of Innsbruck (EK Nr.: 1023/2024) and took place at the traumatological as well as the general and surgical intensive care unit of the Department for Anaesthesiology and Intensive Care Medicine of the Innsbruck Medical University Hospital. Approximately 700–800 mainly surgical (cardiac, visceral, thoracic, transplant, orthopedics and traumatology) but also medical patients are treated annually on 23 level-3 beds, in total.

The Sequential Organ Failure Assessment (SOFA) score and the Simplified Acute Physiology Score III (SAPS3) were applied as part of the generalized propensity score matching method. Both scores are established prognostic tools in critical care medicine. The SOFA score evaluates the severity of organ dysfunction across six systems (respiratory, cardiovascular, hepatic, renal, coagulation, and neurological), while the SAPS3 score predicts hospital mortality using 20 variables, including age, comorbidities, and acute physiological parameters (e.g., Glasgow Coma Scale, blood pressure, laboratory values) collected within the first hour of ICU admission [16,17,18,19,20].

**Figure 1:**
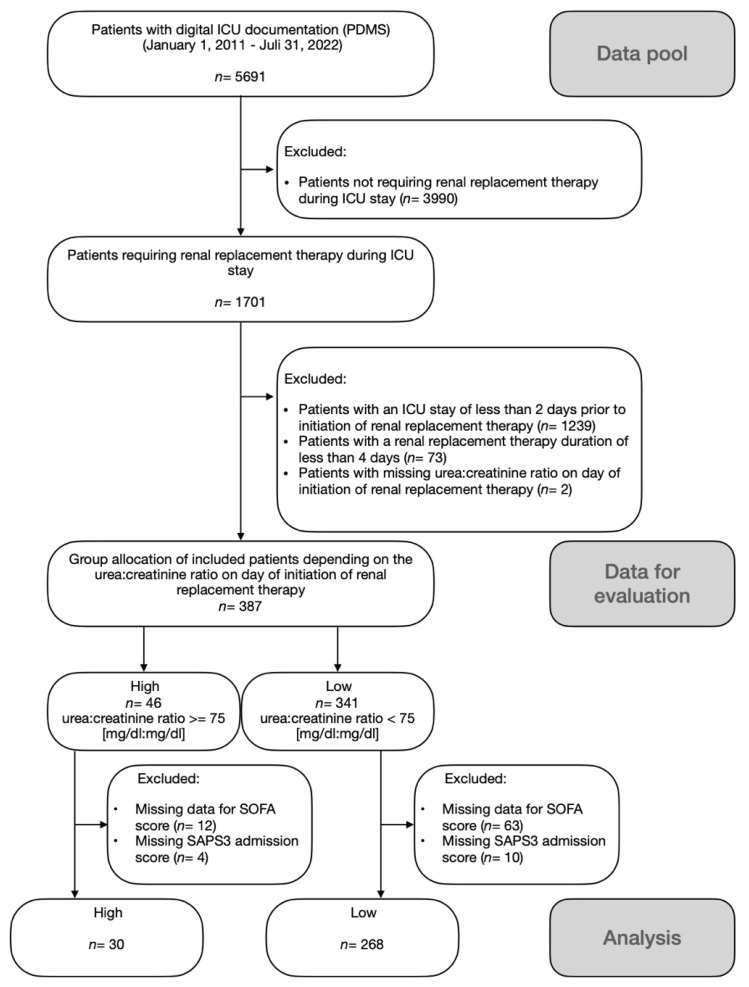
Study flow chart.

Patients with digital ICU documentation in our patient data management system (PDMS) within a 11.5-year timeframe from 1 January 2011 to 31 July 2022 were selected. Digital ICU documentation was implemented on one ICU ward in 2007, while the other ward adopted digital documentation in 2018. The primary data pool comprised 5691 ICU patients (Figure 1). Patients not requiring CRRT at all (n = 3990) and patients with an ICU stay of less than 2 days prior to CRRT initiation (n = 1239), as well as patients with a total CRRT duration of less than 4 days (n = 73), were excluded. Further exclusion criteria were (i) missing UCR on the day of CRRT initiation (n = 2); (ii) missing data for SOFA score (n = 75); and (iii) missing SAPS3 admission score (n = 14). Groups were allocated depending on the height of the UCR before CRRT initiation (high: UCR ≥ 75; low: UCR < 75 [mg/dL:mg/dL]), yielding a high (n = 30) and a low group (n = 268).

In general, CRRT was performed either as continuous venovenous hemofiltration (Prismaflex, Baxter, Deerfield, IL, USA/Gambro, Lund, Sweden) or as continuous venovenous haemodialysis (multiFiltrate, Fresenius Medical Care, Bad Homburg, Germany), depending on the ICU ward where the patient was treated. Regional citrate anticoagulation and a target dialysis dose of 20–30 mL/kg/h are standard practice in our department.

UCR trajectories over time were compared between treatment groups undergoing CRRT and matched control groups not requiring CRRT. A generalized propensity score matching method was applied (GenMatch from the R package: ‘Matching’ [21]), considering age, sex, bodyweight, and SAPS3 admission score, as well as the day of ICU and SOFA score on the day of CRRT start, and the UCR on the day of and the day before CRRT start. Patients were matched 1:1 in the low group and in a 2:1 manner in the high group due to the small sample size. Baseline day 0 was defined as day of CRRT start in the treatment group and the corresponding ICU day for the control groups. The observation period was from two days prior to CRRT initiation to four days after. Due to high rates of sedation on ICU, SOFA scores were calculated without consideration of the Glasgow Coma Scale [22]. Protein and calorie intakes were calculated from the given amounts of enteral- and parenteral nutrition, as well as non-nutritional applications of glucose solutions (e.g., maintenance fluids, drug solvents) and propofol.

Statistical analysis was performed using R (v4.3.2, R Core Team) and RStudio (v2023.12.0-369, RStudio, Inc., Boston, MA, USA). Due to the non-normal distribution (Shapiro–Wilk test), data are presented as count and percentage or median and interquartile range (Q1–Q3). Group differences in frequencies were analyzed via Chi-squared test and group differences in continuous data via Mann–Whitney U test on a day-to-day basis. The graphical presentation of daily trends was conducted via generalized additive models with a 95% confidence interval, utilizing the R package ggplot2.

A *p*-value < 0.05 was considered statistically significant.

## 3. Results

There were no significant differences in age, sex, bodyweight, SAPS3, baseline SOFA score, or UCR values at baseline and the day before when comparing the treatment groups with their matched controls (Table 1). The median UCR at baseline was 93.4 (82.5–105.3) and 41.9 (32.0–53.0), the medians day on ICU were 9 (6–12) and 3 (3–4) in the high and low group, respectively (Table 2). Moreover, no differences were observed between the low and the high groups with respect to sex, age, bodyweight, the utilized CRRT mode (hemofiltration or hemodialysis), or dose (Table 2). While SAPS3 was higher in the high group, the SOFA-Score on baseline day was higher in the low group.

**Figure 2:**
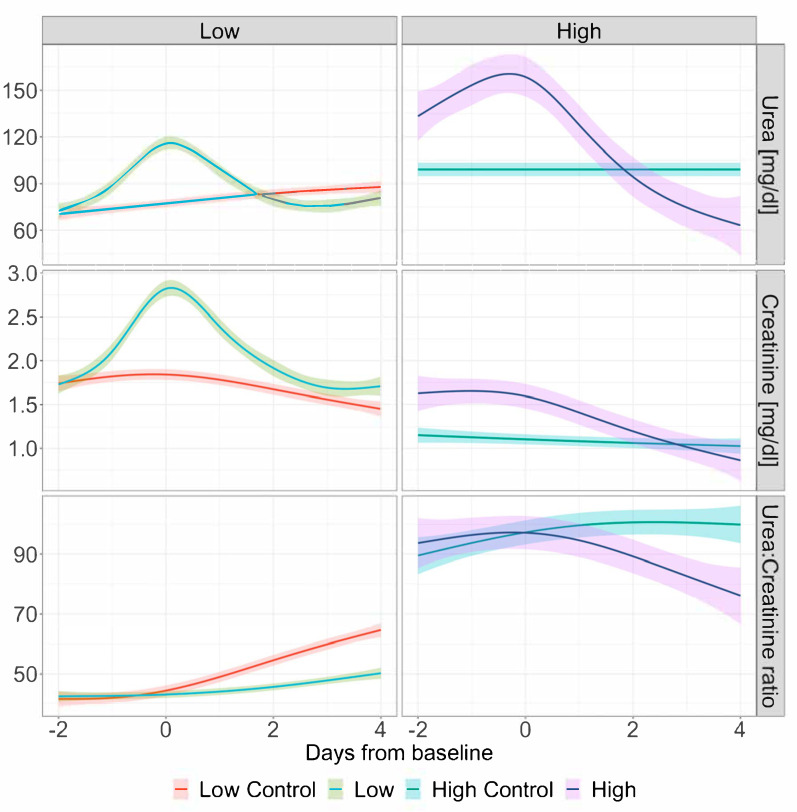
Trends in serum urea, creatinine, and urea–creatinine ratio during the observation period. Graphical presentation via generalized additive models with 95% confidence interval. Baseline day 0 is defined as the day of CRRT initiation in treatment groups and the corresponding day on ICU in matched controls. The low group is defined as an UCR < 75 and the high group as an UCR ≥ 75 on the day of CRRT initiation.

**Figure 3:**
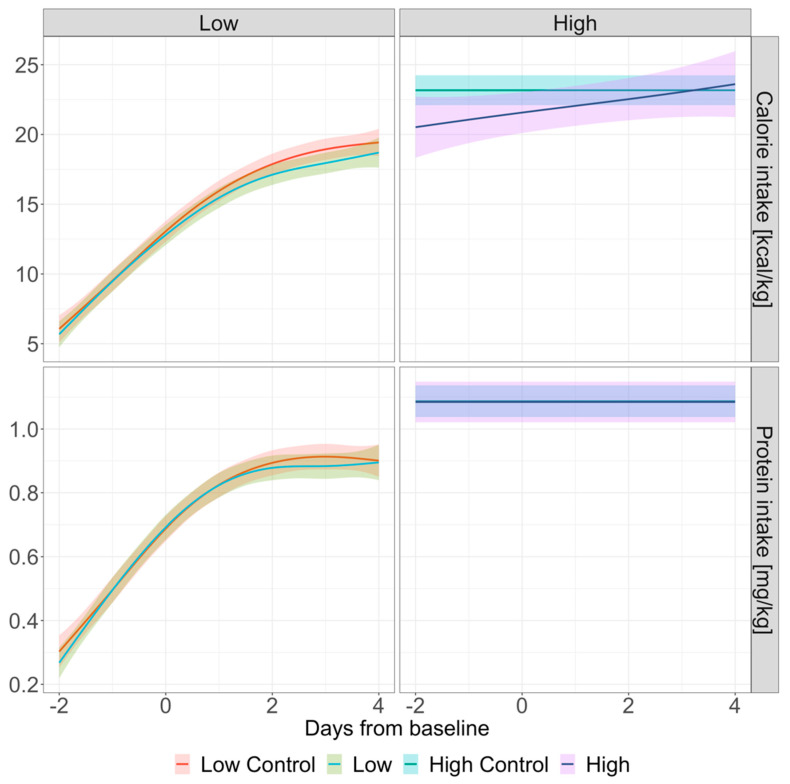
Trends in daily calorie and protein intakes per kg during the observation period. Graphical presentation via generalized additive models with 95% confidence interval. Baseline day 0 is defined as day of CRRT initiation in treatment groups and the corresponding day on ICU in matched controls. The low group is defined as an UCR < 75 and the high group as an UCR ≥ 75 on the day of CRRT initiation.

The day-to-day comparison between matched controls and treatment groups revealed significant differences in the UCR on day 1 (44.0 (34.2–59.8) vs. 40.6 (32.1–52.5) (*p* = 0.024) and thereafter in the low group, and on day 2 (94.4 (83.0–115.2) vs. 85.0 (69.5–96.4); *p* = 0.036) and thereafter in the high group (Table 2, Figure 2). In the high group, on day 3 after baseline, median UCR values fell below the threshold of 75 (74.5 (61.5, 93.0) vs. 98.6 (77.4, 114.4); *p* = 0.001). Calorie and protein intakes remained comparable across the entire observation period (Table 3, Figure 3). As shown in Figure 2, the trends in urea and creatinine differed between the treatment groups, with urea levels tending to be higher in the high group and creatinine levels being higher in the low group at baseline. Trends in calorie and protein intakes over time are illustrated in Figure 3. Since the median baseline day 0 (CRRT start) falls on ICU day 3 in the low group and ICU day 9 in the high group, the observed differences in calorie and protein intakes likely reflect more advanced diet progression in the high group.

## 4. Discussion

In this study, the impact of CRRT on the progression of the UCR was analyzed. Patients requiring CRRT were compared to matched controls based on sex, age, bodyweight, SAPS3 admission score, baseline day 0, day of ICU stay, SOFA score on baseline day, and UCR on baseline day and the day before. The analysis revealed a substantial effect of CRRT on the UCR. Depending on the baseline UCR levels, a significant decrease was observed in the high-UCR group (≥75 mg/dL:mg/dL) during CRRT, while an attenuated increase was seen in the low-UCR group (<75 mg/dL:mg/dL).

We chose the mentioned threshold based on the study by Haines et al., which established the UCR as a surrogate for catabolism. In detail, patients who developed persistent critical illness had a UCR of 141 mmol/L:mmol/L (=75 mg/dL:mg/dL) on ICU day 10, which was significantly higher than those who did not (97 mmoL/L:mmol/L; *p* < 0.001). As described, this was accompanied by increased protein metabolism. We have adopted this threshold as it appears to effectively identify increased protein metabolism, and we applied it in several of our recent studies [4,5,7,23].

Regarding the chosen cutoff for the UCR, there is in fact no established threshold for the UCR yet. As other factors also influence the UCR (dehydration, gastrointestinal bleeding, exogenous dietary protein intake, kidney and liver function, CRRT), patient group-specific thresholds are still warranted [24]. However, it is widely recognized that an elevated UCR is associated with catabolism and general muscle loss [2,3,8,24]

After the matching process, no significant differences were observed in baseline characteristics when comparing the groups to their matched controls. However, when comparing the low- and high-CRRT groups, the inherently higher baseline UCR in the high UCR group resulted in a noticeable difference in the timing of CRRT initiation (baseline day 0). On average, CRRT was initiated on ICU day 3 in the low UCR group, and on ICU day 9 in the high UCR group. This discrepancy is significant because it reflects the different stages of illness and metabolic stress that each group was experiencing at the time of CRRT initiation.

For the patients in the low groups, who were likely still in the initial phase of their ICU stay, a rise in the UCR can be expected due to post-aggression-related increased protein metabolism. As a result, the rise in UCR is particularly evident in the low control group. In contrast, the high control group demonstrates a levelling off of the UCR, indicating the presence of a plateau phase. Given these varying UCR dynamics due to differing baseline values, the impact of CRRT on UCR progression must differ as well. In the low group, CRRT attenuated the rise in UCR, while in the high group, it led to a reduction in UCR levels.

Another significant difference between the high and the low groups is the baseline levels of creatinine and urea. While initial creatinine is higher in the low, initial urea is higher in the high group. This may be attributed to varying CRRT indications that were not factored into the study, potentially influencing the outcomes, e.g., uremic encephalopathy in the high UCR group versus oliguria in the low UCR group, but again, the differing phases of ICU stay may also play a key role. With a probably higher impact of muscle breakdown due to the longer stay in ICU, a relative decrease in creatinine levels and, vice versa, an increase in urea levels can be expected.

Given the central focus of this study—the impact of CRRT on the UCR—it is crucial to closely examine the specific molecules involved.

Both urea and creatinine are water-soluble smaller molecules (60 Da vs. 113 Da) easily filtered by CRRT membranes. The sieving coefficients across the CRRT membrane are quite similar, lying in the range of 23–38 mL/min for urea and 26–43 mL for creatinine [25]. Given these comparable elimination rates, the UCR should be valid under ongoing CRRT as well. However, on the other side of elimination of course stands production and the uncertainty concerning consistent clearance rates throughout the full filter life span of 72 h. In the context of overproduction, elimination might not be able to follow, potentially leading to an accumulation despite ongoing efforts to clear the excess. Depending on the patient’s underlying total urea production rate, the UCR can still rise despite CRRT, albeit to a lesser extent, as seen in the low group in this study.

Median UCRs went below the threshold of 75 mg/dL:mg/dL on the third day after CRRT initiation in the high group, thereby falling short to further distinguish ongoing catabolism. Importantly, an elevated UCR is not only influenced by increased muscle breakdown. An elevated protein metabolism through high protein intake will also lead to an increased urea production rate and subsequently to an elevated UCR [26]. The UCR cannot distinguish if extrinsic or intrinsic protein is being metabolized. Additionally, gastrointestinal bleeding can also lead to an elevated UCR, again through high protein metabolism by the digestion of blood [9,27]. In heart failure, an elevated UCR is thought to reflect neurohumoral activation or altered renal blood flow rather than catabolism and is associated with poorer outcome [28,29,30]. The overall impact of kidney function on UCR remains poorly understood. Initially, it was thought that the UCR might be independent of renal function as both urea and creatinine are excreted renally. The assumption was that an impaired renal function would lead to a decrease in both urea and creatinine elimination, not affecting the UCR [31]. Again, depending on relative elimination rates, the UCR might increase with failing renal function [32,33], or even decrease in severe AKI, especially in patients with acute interstitial nephritis [34].

Since increased protein metabolism (whether extrinsic or intrinsic) remains the primary factor contributing to an elevated UCR, it is important to highlight that besides having similar disease severity based on SAPS3 admission scores and SOFA scores, both CRRT groups and controls also had comparable calorie and protein intakes throughout the observation period, ensuring good overall comparability between the groups.

The main limitations of our study are the single-centre retrospective design and the different group sizes regarding high and low UCR. However, we do believe that our main conclusions remain robust, as CRRT groups were not only compared to each other but to groups of matched controls not requiring CRRT.

## 5. Conclusions

The UCR is notably impacted by continuous renal replacement therapy. Depending on the baseline UCR levels, patients experienced either a decrease or a more gradual increase in UCR compared to their matched controls.

This suggests that CRRT plays a significant role in modulating UCR, with its effects varying according to the patients’ initial metabolic state. Patients with higher baseline UCR levels experienced a significant reduction, while those with lower baseline levels showed an attenuated increase. This indicates that the impact of CRRT on UCR is likely influenced by the severity of protein catabolism at the onset of therapy.

## Figures and Tables

**Table 1:** General demographics and matching variables for low group vs. low controls and high group vs. high controls.

	Low Control (n = 268) n (%) or Median (Q1, Q3)	Low (n = 268) n (%) or Median (Q1, Q3)	*p*	High Control (n = 60) n (%) or Median (Q1, Q3)	High (n = 30) n (%) or Median (Q1, Q3)	*p*
**Sex**			1.000			1.000
**Female**	71 (26.5%)	71 (26.5%)		14 (23.3%)	7 (23.3%)	
**Male**	197 (73.5%)	197 (73.5%)		46 (76.7%)	23 (76.7%)	
**Age [years]**	70.0 (62.8, 76.0)	70.5 (60.8, 77.0)	0.916	70.0 (59.0, 74.3)	69.0 (63.0, 75.0)	0.523
**Bodyweight [kg]**	76.0 (69.7, 87.9)	76.0 (67.4, 88.8)	0.795	71.9 (64.5, 84.0)	70.0 (62.8, 83.9)	0.768
**SAPS 3 ^+^**	64.5 (56.0, 75.0)	64.5 (56.0, 76.0)	0.986	72.0 (63.8, 85.0)	72.5 (62.0, 82.0)	0.881
Baseline day 0 * **[as day on ICU]**	3.0 (3.0, 4.0)	3.0 (3.0, 4.0)	0.654	10.0 (7.0, 12.0)	9.0 (6.0, 11.8)	0.460
**SOFA-Score ^#^** **on day 0**	9.0 (8.0, 11.0)	10.0 (8.0, 11.0)	0.441	8.0 (6.0, 10.0)	8.0 (6.3, 10.0)	0.534
**Urea–creatinine ratio on day 0 [mg/dL:mg/dL]**	39.9 (31.0, 53.5)	41.9 (32.0, 53.0)	0.998	90.7 (81.8, 101.6)	93.4 (82.5, 105.3)	0.706
**Urea–creatinine ratio on day −1 [mg/dL:mg/dL]**	38.5 (31.6, 48.0)	40.5 (31.9, 50.0)	0.361	88.1 (79.9, 101.8)	89.1 (80.3, 106.4)	0.653

^+^ Simplified Acute Physiology Score 3. * baseline day 0 defined as day of CRRT initiation in treatment groups and corresponding ICU day in controls. ^#^ Sequential Organ Function Assessment.

**Table 2:** General demographics for low vs. high group.

	Low (n = 268) n (%) or Median (Q1, Q3)	High (n = 30) n (%) or Median (Q1, Q3)	*p*
**Sex**			0.709
**Female**	71 (26.5%)	7 (23.3%)	
**Male**	197 (73.5%)	23 (76.7%)	
**Age [years]**	70.5 (60.8, 77.0)	69.0 (63.0, 75.0)	0.999
**Bodyweight [kg]**	76.0 (67.4, 88.8)	70.0 (62.8, 83.9)	0.077
**SAPS 3 ^+^**	64.5 (56.0, 76.0)	72.5 (62.0, 82.0)	0.029
Baseline day 0 * **[as day on ICU]**	3.0 (3.0, 4.0)	9.0 (6.0, 11.8)	<0.001
**SOFA-Score ^#^** **on day 0**	10.0 (8.0, 11.0)	8.0 (6.3, 10.0)	0.005
**Urea–creatinine ratio on day 0 [mg/dL:mg/dL]**	41.9 (32.0, 53.0)	93.4 (82.5, 105.3)	<0.001
**Urea–creatinine ratio on day −1 [mg/dL:mg/dL]**	40.5 (31.9, 50.0)	89.1 (80.3, 106.4)	<0.001
**Mode of CRRT ^$^**			0.893
**hemofiltration**	178 (66.4%)	19 (63.3%)	
**hemodialysis**	90 (33.6%)	11 (36.7%)	
**Dose of CRRT**			
**Blood flow** **[ml/min]**	100.0 (100.0, 114.6)	100.0 (99.5, 120.7)	0.877
**Effluent rate** **[ml/kg/h]**	21.1 (15.1, 30.1)	24.2 (16.1, 33.9)	0.392

^+^ Simplified Acute Physiology Score 3. * baseline day 0 defined as day of CRRT initiation. ^#^ Sequential Organ Function Assessment. ^$^ continuous renal replacement therapy.

**Table 3:** Daily comparison between treatment groups and matched controls regarding the urea–creatinine ratio, calorie intake, and protein intake per kg during the observation period.

	Day −2	Day −1	Day 0	Day 1	Day 2	Day 3	Day 4
**Urea–Creatinine ratio**							
Low Control	38.7 (32.9, 49.3)	38.4 (31.5, 47.5)	39.9 (31.0, 53.5)	44.0 (34.2, 59.8)	49.0 (39.1, 68.4)	55.4 (42.7, 72.1)	59.8 (45.1, 78.9)
Low	40.3 (31.2, 49.6)	40.5 (31.9, 50.0)	41.9 (32.0, 53.0)	40.6 (32.1, 52.5)	42.1 (33.2, 52.5)	43.9 (36.5, 54.8)	47.2 (38.5, 58.1)
*p*	0.787	0.311	0.998	0.024	<0.001	<0.001	<0.001
High Control	84.7 (64.3, 101.6)	88.1 (79.9, 101.8)	90.7 (81.8, 101.6)	95.4 (83.2, 114.3)	94.4 (83.0, 115.2)	98.6 (77.4, 114.4)	92.6 (74.8, 108.1)
High	84.0 (75.9. 106.5)	89.1 (80.3, 106.4)	93.4 (82.5, 105.3)	89.3 (78.0, 101.3)	85.0 (69.5, 96.4)	74.5 (61.5, 93.0)	70.4 (56.0, 88.1)
*p*	0.635	0.656	0.710	0.453	0.036	0.001	0.005
**Calorie intake [kcal/kg]**							
Low Control	1.8 (0.2, 10.8)	8.6 (1.7, 14.0)	12.0 (7.8, 17.4)	15.4 (11.0, 20.4)	17.0 (12.3, 21.7)	18.1 (14.0, 22.6)	18.4 (13.3, 24.0)
Low	1.3 (0.2, 6.9)	7.4 (2.3, 13.4)	11.1 (8.1, 17.4)	14.9 (10.5, 19.9)	17.0 (12.4, 22.1)	17.6 (12.8, 22.5)	18.3 (14.1, 23.2)
*p*	0.158	0.947	0.470	0.501	0.711	0.143	0.708
High Control	21.0 (14.0, 32.5)	20.0 (15.4, 32.5)	20.7 (16.3, 30.9)	21.7 (17.0, 30.4)	23.4 (16.3, 29.5)	22.2 (18.4, 29.7)	23.7 (18.2, 30.9)
High	17.8 (13.6, 24.6)	21.0 (15.6, 26.8)	21.7 (16.6, 27.2)	21.7 (17.7, 26.8)	20.9 (17.9, 27.8)	21.7 (15.7, 27.4)	24.5 (20.4, 34.2)
*p*	0.144	0.942	0.969	0.725	0.535	0.613	0.368
**Protein intake [g/kg]**							
Low Control	0.0 (0.0, 0.6)	0.4 (0.0, 0.8)	0.7 (0.4, 0.9)	0.8 (0.6, 1.1)	0.9 (0.6, 1.1)	0.9 (0.7, 1.1)	0.9 (0.6, 1.2)
Low	0.0 (0.0, 0.4)	0.4 (0.1, 0.7)	0.7 (0.4, 0.9)	0.8 (0.6, 1.0)	0.9 (0.6, 1.1)	0.9 (0.6, 1.1)	0.9 (0.7, 1.1)
*p*	0.128	0.970	0.962	0.832	0.736	0.241	0.856
High Control	1.0 (0.8, 1.4)	1.0 (0.8, 1.4)	0.9 (0.8, 1.5)	1.0 (0.8, 1.4)	1.0 (0.8, 1.4)	1.0 (0.8, 1.3)	1.0 (0.7, 1.4)
High	0.9 (0.7, 1.1)	1.1 (0.9, 1.3)	1.0 (0.9, 1.2)	1.0 (0.8, 1.3)	1.0 (0.8, 1.3)	1.0 (0.8, 1.4)	1.0 (0.9, 1.5)
*p*	0.274	0.758	0.635	0.845	0.968	0.891	0.550

## Data Availability

The data that support the findings of this study are not publicly available due to containing information that could compromise the privacy of research participants but are available from the corresponding author (P.H.) upon reasonable request.

## Abbreviations

The following abbreviations are used in this manuscript:

UCR	urea–creatinine ratio
CRRT	continuous renal replacement therapy
ICU	intensive care unit
AKI	acute kidney injury
SOFA	sequential organ failure assessment score
SAPS3	simplified acute physiology score 3
PDMS	patient data management system
